# Glycated Hemoglobin (HbA1c) Concentrations Among Children and Adolescents With Diabetes in Middle- and Low-Income Countries, 2010–2019: A Retrospective Chart Review and Systematic Review of Literature

**DOI:** 10.3389/fendo.2021.651589

**Published:** 2021-04-12

**Authors:** Xiuli Chen, Zhou Pei, Miaoying Zhang, Zhenran Xu, Zhuhui Zhao, Wei Lu, Linqi Chen, Feihong Luo, Ting Chen, Chengjun Sun

**Affiliations:** ^1^Department of Endocrinology, Genetics and Metabolism, Children’s Hospital of Soochow University, Suzhou, China; ^2^Department of Pediatric Endocrinology and Inherited Metabolic Diseases, Children’s Hospital of Fudan University, Shanghai, China

**Keywords:** glycemic control, diabetes mellitus, childhood, HbA1c, middle- and low-income country

## Abstract

**Objectives:**

To explore the glycemic control [represented by glycated hemoglobin (HbA1c) concentrations] in children with diabetes mellitus (DM) in east China and middle- and low-income countries, from 2010 to 2019.

**Methods:**

Retrospective data of children with DM from two hospital-based health records were reviewed. Data on HbA1c concentrations, hospitalization due to diabetic ketoacidosis, and patient demographics were collected and analyzed. A systematic review was subsequently performed to analyze publications that report HbA1c concentrations in patients aged <18 years. Patients’ characteristics extracted from each publication were used to generate simulated individual data for pooled analysis. HbA1c estimates were derived from steady-state iterations.

**Results:**

Data of 843 diabetic children (aged 11.2 ± 3.9 years) with 2,658 HbA1c measures were retrieved from the two hospitals during the period 2010–2020. The duration of diabetes in the patients was 4.4 ± 2.8 years, and their HbA1c was 8.1 ± 2.2%. Patients who were internal migrants had significantly higher HbA1c concentration than resident patients (8.4 *vs.* 7.9%). The literature review yielded 1,164 publications, and the majority (74.1%) of patient data were published in high-income countries. The patient data extracted from these publications generated 486,416 HbA1c concentration estimates between 2005 and 2019. The average HbA1c concentration during the 15 years was 9.07 ± 2.15%. The mean HbA1c concentrations among children were 8.23, 8.73, 9.20, and 10.11% in high-income country (HIC), upper-middle income country (UMIC), lower-middle income country (LMIC), and low-income country (LIC) respectively. The mean rate of optimized glycemic control (HbA1c <7.5%) among children was 32.4, 27.5, 21.7, and 12.7% in HIC, UMIC, LMIC, and LIC, respectively.

**Conclusions:**

The current study indicated that there is substantial room for improvement in glycemic control in children with DM worldwide, especially in middle- and low-income countries.

## Introduction

Diabetes mellitus (DM) in childhood is estimated to affect about 0.4/1,000 of the worldwide children’s population (1). DM in children is primarily (>80%) type 1 diabetes mellitus (T1DM), with a minor fraction having type 2 diabetes mellitus (T2DM) (2). The incidence of childhood diabetes varies among populations. In children aged <15 years, T1DM occurs with an annual incidence of 5–50 per 100,000 persons (3), while the incidence of T2DM incidence is associated with a relatively larger variation (4). DM is a chronic disease that requires life-long management. Recent population-based studies have shown that in countries with high-income, the average loss of life expectancy in patients with T1DM is about 12–15 years owing to diabetes-related complications (5, 6). The childhood-onset DM is associated with premature death, especially among patients with higher glycated hemoglobin (HbA1c) concentrations (7).

In the 2000s, results from the Diabetes Control and Complications Trial/Epidemiology of Diabetes Interventions and Complications (DCCT/EDIC) (8) and the United Kingdom Prospective Diabetes Study (UKPDS) (9) demonstrated that stringent glycemic control, as indicated by lower HbA1c concentrations, resulted in significantly less chronic diabetic complications. Recent population-based studies in adults confirmed that higher HbA1c concentration is associated with increased diabetic complications (10) and complication-related mortality (11). Despite that HbA1c concentration can be influenced by many factors from childhood to adulthood (12), there is also a clear association between lower HbA1c during childhood and less diabetic complications in adulthood (13). In the past few years, international guidelines have adopted stricter HbA1c goals as compared to their previous recommendations for DM in children and adolescents: The American Diabetes Association (ADA) recommended HbA1c <7.5% in 2015 (14), the National Institute for Health and Care Excellence (NICE) recommended HbA1c <6.5% in 2016 (15), and the International Society for Pediatric and Adolescent Diabetes (ISPAD) recommended HbA1c <7.0% for children with DM in 2018 (16).

Many factors can influence the HbA1c in a child with DM, for instance, the patient’s age, duration of illness, access to healthcare resources, relationship between family members, and social-economic status of his/her family (17, 18). Recent large-scale population-based cohorts in children with DM showed that the average HbA1c concentration is higher than the <7.5% cut-off in nearly all high-income countries (19). Meanwhile, studies on HbA1c concentrations in children and adolescents are sparse in middle- and low-income countries where patients have less access to healthcare resources and are thus at a higher risk of poor glycemic control (20). Therefore, in the current study, we analyzed patient records of children with DM in two children’s hospitals in east China and conducted a systematic review to analyze the concentrations of HbA1c among children and adolescents using data from previous publications.

## Patients and Methods

### Patients

Patients included in the current study were children with DM in two hospitals (Children’s Hospital of Fudan University in Shanghai and Children’s Hospital of Soochow University in Suzhou). Both hospitals are tertiary pediatric hospitals in east China. The gross domestic product (GDP) per capita in 2010–2019 for Shanghai and Suzhou were $11,230–22,800 and $21,450–26,000, respectively (21, 22). All patients from the two hospitals with a diagnosis of DM in their electronic health records were included for retrospective chart review. The DM diagnosis and classification were confirmed according to the 2010 ADA criteria (23). We extracted data of each patient’s hospital visit for statistical analysis, including demographics (patient’s date of birth, sex, household registration address); date of hospital visit; duration of illness; type of diabetes; HbA1c concentrations (>1 year after diagnosis); and hospital visit owing to diabetic ketoacidosis. Patients were grouped as residents and migrants based on whether their household registration address and the hospital were in the same city. Patients aged 0–18 years at the time of DM diagnosis were included. Data included in the current study were collected from January 2010 to September 2020. We report our patients as recommended by STROBE (24).

### Systematic Review

The literature search was performed in Ovid MEDLINE (All database, 1946 to present) to identify published studies on children and adolescents with DM between January 2010 and November 2020 (last search performed on December 1, 2020; **Supplemental Table 1**). The following inclusion criteria were used: 1) subjects were children with DM aged between 0 and 18 years; and 2) the “baseline” HbA1c concentration was reported before any intervention (*i.e.*, clinical trial or prospective follow-up). The exclusion criteria were: 1) publications that did not report HbA1c concentrations (*e.g.*, comments, editorials, reviews, protocols, guidelines, studies on pre-diabetic or healthy individuals, studies on healthcare policy or environment, and studies on non-human subjects); 2) studies in adults (*e.g.*, gestational DM, DM in parents); 3) HbA1c was reported with possible selection bias [*e.g.*, studies with predefined HbA1c concentration in patient selection, studies among subjects with diabetic complications; studies on newly diagnosed (<1 year) DM patients]; 4) case reports or studies on monogenic DM; and 5) publications that were not written in English or Chinese. After the search, the titles and abstracts of publications were independently screened by two authors (XC and CS), and any discrepancies were reviewed by a third author (PZ) and resolved by discussion.

Data were extracted from each included study on the general information (year of publication, and country of the studied subjects) and the following baseline characteristics of the studied patients: sample size of the study, type of diabetes (T1DM or T2DM), age [mean, standard deviation (SD) and range], sex (male proportion), duration of diabetes (mean, SD and range), HbA1c concentration (mean, SD and range), and year of data collection. All publications were matched by the country and authors’ names and manually reviewed to eliminate duplicated reports from an identical patient cohort. The systematic review was performed in accordance with the PRISMA guideline (25).

### Statistical Methods

The data analysis was performed using R (version 4.0.3, www.r-project.org). The summary statistics of data from our patients are presented as mean ± SD. The values of each HbA1c measurement were independently pooled as cross-sectional data in the statistical analysis. The comparison between resident and migrant patients was conducted using Fisher’s exact test for proportional data and unpaired *t*-test or Wilcoxon rank sum test for continuous variables with *α* = 0.05 as the cut-off for statistical difference. The trend of HbA1c change over the calendar year was assessed using linear regression.

For data analysis from the systematic review, the simulated individual patient’s characteristics (HbA1c, age, and duration of diabetes) were generated assuming normal distribution in each characteristic using parameters (mean, SD, and range) according to the sample size reported in each publication. The remaining characteristics (sex, type of diabetes, and calendar year) were randomly generated according to data provided by each publication. Missing parameters in each publication were imputed by the distribution of the available data (HbA1c, age, duration of diabetes, gender, and duration of study) from other publications with coefficients assuming linear regression between the mean and the other parameters (SD, range, and year of publication). After the above characteristic simulates were randomly assigned with each other to form simulates of patients representing each published study, the simulated patients of all studies were then pooled by subgroups (types of diabetes, sex, age, duration of diabetes, and calendar year), to generate HbA1c estimates [arithmetic mean and SD and their 95% confidence intervals (CI)] from steady-state iterations. The difference in HbA1c between groups was compared by two-tailed unpaired *t*-test based on the estimated parameter of the HbA1c distribution and effect size (Cohen’s *d*) based on differences between means. P-value <0.05 and effect size >0.2 were considered to indicate significant difference (26). In addition to the main analysis, we conducted sensitivity analysis by excluding large population-based studies and studies with incomplete parameters of patients’ characteristics (age, duration of diabetes, calendar year, or sex).

The countries of studied subjects in each publication were grouped by income [*i.e.*, high-income countries (HIC), upper-middle-income countries (UMIC), lower-middle-income countries (LMIC), and low-income countries (LIC)] in each year according to the World Health Organization/World Bank income classification (27).

### Ethics

The studies involving human participants were reviewed and approved by the Faculty Hospital Ethics Committee in Children’s Hospital of Fudan University, and the Ethics Committee of Children’s Hospital of Soochow University. Written informed consent to participate in this study was provided by the participants’ legal guardian/next of kin.

## Results

### Glycemic Control Among Children and Adolescents With Diabetes in East China

Between 2010 and 2019, we identified 843 pediatric patients with duration of DM longer than 1 year and 2,658 HbA1c measures in our hospitals. Among the patients, 407 (48.3%) were male and 791 (93.8%) were diagnosed with T1DM. The average age of patients during the 10 years was 11.2 ± 3.9 years, and the duration of diabetes was 4.4 ± 2.8 years. The average follow-up duration was 2.1 ± 2.0 years. During their visits, HbA1c was measured 1.8 ± 1.3 times per year in our patients. During the study period, the patients’ HbA1c was 8.1 ± 2.2%. The incidence of diabetic ketoacidosis (DKA) in our patients was 25 per 1,000 patient–years (**Table 1**). Patients with T1DM had significantly higher HbA1c than patients with T2DM (8.1 *vs.* 7.7%, P < 0.001). The HbA1c concentrations were not different between sexes, and HbA1c concentrations increased with age (**Table 1**).

**Table 1:** HbA1c in childhood DM patients in east China.

	All	Patient type		P-value^*^
		Resident	Migrant	
Number of patients/HbA1c measures	843/2658	403/1473	440/1185	
T1DM, N (%)	791 (93.8%)	373 (92.6%)	418 (95%)	0.62
Age	11.2 ± 3.9	11.3 ± 3.8	11.1 ± 4	0.049
Male, N (%)	407 (48.3%)	191 (47.4%)	216 (43.4%)	0.81
Duration of diabetes	4.4 ± 2.8	4.5 ± 2.8	4.2 ± 2.8	<0.001
Follow-up duration	2.1 ± 2.0	2.3 ± 2.1	2.0 ± 1.9	<0.001
Number of HbA1c measures per patient	3.2 ± 3.3	3.7 ± 3.8	2.7 ± 2.6	<0.001
Number of HbA1c measures per patient-year	1.8 ± 1.3	1.8 ± 1.1	1.7 ± 1.4	0.016
HbA1c	8.1 ± 2.2	7.9 ± 2	8.4 ± 2.4	<0.001
Proportion of HbA1c <7.5%	1267 (47.7%)	759 (51.5%)	508 (42.9%)	
Proportion of HbA1c ≥9.0%	685 (25.8%)	304 (20.6%)	381 (32.2%)	
DKA events, total events (events per 1,000 patient-years)	45 (25)	18 (20)	27 (31)	
*HbA1c in subgroups*				
by diabetes type				
T1DM	8.1 ± 2.2	7.9 ± 2.0	8.4 ± 2.4	<0.001
T2DM	7.7 ± 2.8	7.4 ± 2.6	8.1 ± 3.0	0.13
by sex				
Male	8.1 ± 2.2	7.8 ± 1.9	8.4 ± 2.4	<0.001
Female	8.2 ± 2.3	8.0 ± 2.1	8.4 ± 2.4	0.002
by age				
0-3y	7.8 ± 1.6	7.5 ± 1.1	7.9 ± 1.8	0.86
3-9y	7.6 ± 1.6	7.4 ± 1.3	7.8 ± 1.9	0.11
9-15y	8.3 ± 2.3	8.0 ± 2.1	8.6 ± 2.5	<0.001
15-18y	8.8 ± 2.8	8.6 ± 2.7	9.2 ± 2.9	0.02
by year				
2010-2014	8.5 ± 2.4	8.3 ± 2.2	8.7 ± 2.5	0.005
2015-2019	8.0 ± 2.2	7.8 ± 2.0	8.2 ± 2.4	0.004

Data are presented in mean ± standard deviation, DM, diabetes mellitus; HbA1c, glycated hemoglobin; * comparison between resident and migrant patients.

**Figure 1:**
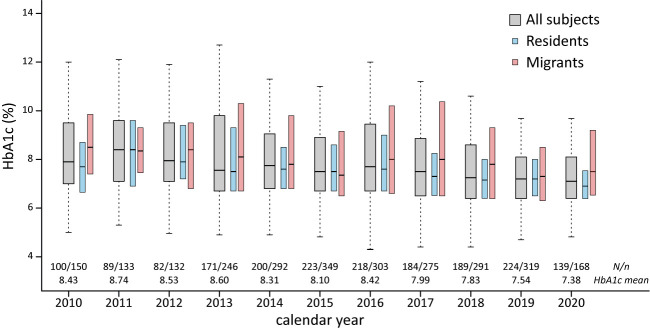
HbA1c among pediatric DM patients in east China, HbA1c, glycated hemoglobin; N, number of subjects/number of HbA1c measures.

After dividing patients according to their address of household registration, we identified that local resident patients had a longer duration of follow-up, more HbA1c measurements, and lower incidence of DKA. The HbA1c concentration was higher among migrant patients than in resident patients (8.4 *vs.* 7.9% respectively). The difference in HbA1c between residents and migrants was observed in patients with T1DM, and older patients (age 9–18 years; **Table 1**). The mean HbA1c concentrations in our patients significantly decreased from year 2010 to 2020 at an annual rate of 0.12% (**Figure 1** and **Supplemental Figure 1**).

### Glycated Hemoglobin in Children and Adolescents With DM in the Literature

**Figure 2:**
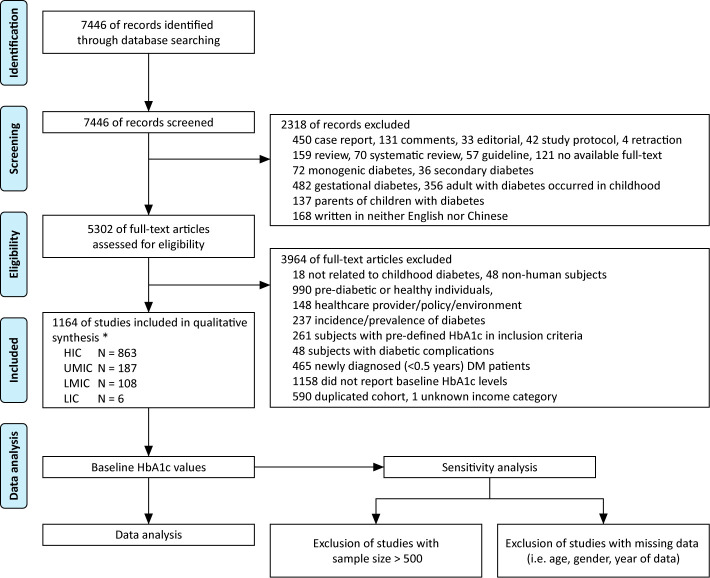
Flow diagram of literature review, * based on 2015 WHO/IMF income category, HIC, high-income country; UMIC, upper middle-income country; LMIC, lower middle-income country; LIC, low-income country; N, number.

**Figure 3:**
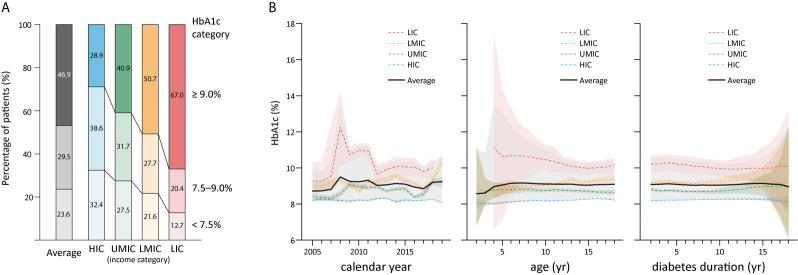
The overall distribution of HbA1c in children by country income level in 2005–2019 **(A)**; and HbA1c in childhood diabetes by year and age, and duration **(B)**, shaded sections indicate 95% uncertainty intervals, HIC, high-income country; UMIC, upper middle-income country; LMIC, lower middle-income country; LIC, low-income country; N, number; HbA1c, glycated hemoglobin; yr, year.

The literature search generated 7,446 publications, and 1,164 were included in the analysis (**Figure 2**). These 1,164 studies yielded a population of 677,415 children and adolescents with DM. The majority of studies were conducted in HICs; there were 863, 187, 108, and six studies in HICs, UMICs, LMICs, and LICs (per the 2015 categorization), respectively. Owing to the substantial amount of data between 2005 and 2009 in the studies, we extended our data analysis to the year 2005–2019. Data extracted from these studies generated a simulated patient population of 486,416 (95% CI 481,031–489,129) between 2005 and 2019. Among these patients, 93.9% was reported by studies conducted in HICs, whereas only 0.3% were descriptions of patients from LICs (**Table 2**). The majority (98.9%) of patients’ diagnosis was T1DM, and nearly half (48.7%) were male patients (detailed numbers of estimates are shown in **Supplemental Table 2**). The average HbA1c concentrations from published studies are 8.23% in HIC, 8.73% in UMIC, 9.20% in LMIC, and 10.11% in LIC, and the HbA1c increased significantly along with the decreased income category. The proportion of patients who reached the goal of HbA1c <7.5% was 32.4% in HIC, 27.5% in UMIC, 21.7% in LMIC, and 12.7% in LIC (**Table 2** and **Figure 3A**). The world average HbA1c in pediatric DM patients (arithmetic mean calculated from these data) was 9.09 ± 2.12%. On average, there were 23.6% (95% CI 23.1–24.1%) children who had good glycemic control with HbA1c <7.5%, and 46.9% (95%CI 46.0–47.7%) children with DM had HbA1c ≥9.0%.

**Table 2:** Estimated HbA1c concentrations of childhood DM patients using data from published studies between 2005 and 2019.

	Average across income categories*	By income			
		HIC	UMIC	LMIC	LIC
N	486,416 (481,031–489,129)	456,793 (451,207–459,370)	18,739 (17,928–19,580)	9,408 (9,129–9,742)	1,430 (1,148–1,626)
Male (%)	48.7% (48.0–49.4%)	51.3% (51.1–51.5%)	47.7% (46.9–48.6%)	47.9% (46.8–49.1%)	48.0% (45.9–50.1%)
Average HbA1c	9.07 ± 2.15	8.23 ± 1.55	8.73 ± 1.98	9.20 ± 2.14	10.11 ± 2.35
Proportion of patients with HbA1c < 7.5%	23.6% (23.1–24.1%)	32.4% (32.3–32.6%)	27.5% (26.8–28.3%)	21.7% (20.9–22.6%)	12.7% (11–14.7%)
Proportion of patients with HbA1c ≥ 9.0%	46.9% (46–47.7%)	28.9% (28.8–29.1%)	40.9% (39.9–42%)	50.7% (49.6–51.7%)	66.9% (64–70%)
Average HbA1c in subgroups					
* by diabetes type*					
* *T1DM	9.08 ± 2.15	8.22 ± 1.55	8.73 ± 1.98	9.20 ± 2.14	10.13 ± 2.36
* *T2DM	8.83 ± 2.05	8.68 ± 2.45	8.81 ± 2.27	8.78 ± 1.58	9.11 ± 1.71
* by sex*					
* *Male	9.06 ± 2.09	8.22 ± 1.55	8.69 ± 1.96	9.18 ± 2.15	10.15 ± 2.36
* *Female	9.13 ± 2.14	8.23 ± 1.56	8.76 ± 2.00	9.21 ± 2.12	10.08 ± 2.34
* by age*					
* *1–<3y	8.90 ± 1.92	8.02 ± 1.52	8.75 ± 1.87	8.79 ± 1.76	9.82 ± 1.97
* *3–<9y	9.18 ± 2.26	8.12 ± 1.51	8.78 ± 1.96	8.95 ± 2.03	10.64 ± 2.50
* *9–<15y	9.12 ± 2.14	8.21 ± 1.53	8.74 ± 1.97	9.19 ± 2.13	10.19 ± 2.38
* *15–<18y	9.05 ± 2.12	8.262 ± 1.60	8.66 ± 2.02	9.34 ± 2.17	10.07 ± 2.32
* by diabetes duration*					
* *1–<6y	9.08 ± 2.20	8.21 ± 1.54	8.74 ± 1.99	9.19 ± 2.15	10.21 ± 2.53
* *6–<12y	9.02 ± 2.09	8.25 ± 1.57	8.73 ± 1.97	9.23 ± 2.12	10.00 ± 2.17
* *12–<18y	9.12 ± 2.03	8.22 ± 1.61	8.98 ± 1.97	9.31 ± 1.94	10.00 ± 1.96
* by calendar year*					
* *2005–2009	8.97 ± 2.07	8.21 ± 1.52	8.55 ± 1.82	9.11 ± 2.09	10.14 ± 2.32
* *2010–2014	9.16 ± 2.14	8.23 ± 1.55	8.87 ± 2.02	9.19 ± 2.19	10.30 ± 2.21
* *2015–2019	9.10 ± 2.02	8.19 ± 1.57	8.85 ± 1.98	9.40 ± 2.03	9.97 ± 2.04

Data are presented in mean ± standard deviation or mean (95% confidence interval), DM, diabetes mellitus; HIC, high-income country; UMIC, upper middle-income country; LMIC, lower middle-income country; LIC, low-income country; N, number; HbA1c, glycated hemoglobin; T1DM, type 1 diabetes mellitus; T2DM type 2 diabetes mellitus; NA, not available; * arithmetic mean (detailed number of patient estimates are shown in **Supplemental Table 2**).

**Figure 4:**
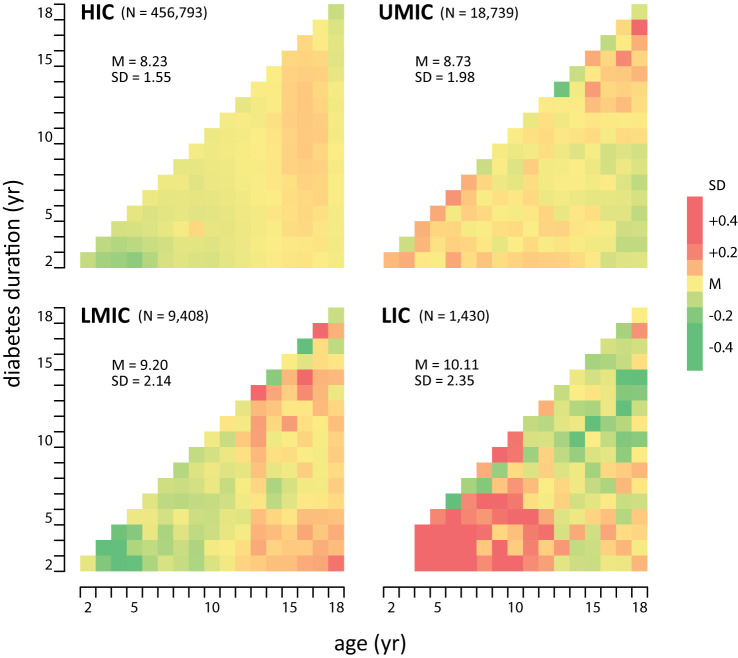
Relative HbA1c concentrations in countries by income classification, The mean HbA1c concentration in each cell corresponds to patients’ age and diabetes duration is represented in a color scale mapping deviation (magnitude of standard deviation) from the country subgroup mean HbA1c, HIC, high-income country; UMIC, upper middle-income country; LMIC, lower middle-income country; LIC, low-income country; N, (mean) numbers of patient estimates; M, mean (of HbA1c); SD, standard deviation (of HbA1c).

The average HbA1c was 9.08% in children with T1DM and 8.83% in those with T2DM (**Table 2**). In patients with T1DM, HbA1c significantly increased from 8.22% in HIC to 10.15% in LIC, whereas the HbA1c in T2DM was not significantly different between adjacent income categories. No significant differences were identified in HbA1c concentrations with respect to sex, age, duration of diabetes, and calendar years (**Table 2**). The mean HbA1c by calendar year, age, and duration of diabetes are plotted in **Figure 3B**. There is a trend that average HbA1c starts to increase in children after age 3–6 years; however, no apparent trend in HbA1c could be observed with respect to calendar years and diabetes duration. The pattern of HbA1c distribution is different among HIC, UMIC, LMIC, and LIC (**Figure 4**). The patient population in HIC showed less fluctuation in HbA1c, and the highest HbA1c can be noted during later puberty (age 14–17 years). However, patients with longer duration in UMIC, older patients with longer diabetes duration in LMIC, and younger patients with shorter duration in LIC had highest HbA1c concentrations (indicated by red color) in each income category (**Figure 3**). After excluding studies that could have caused a deviation of the results, our sensitivity analysis showed similar results to the main analysis (**Supplemental Table 3**).

## Discussion

In the current study, using retrospective hospital records, we observed that the glycemic control of children with DM was improved in our hospitals (in east China) during the last decade. In addition, the migration status of the family was associated with worse glycemic control in diabetic children. The published studies in the last decade show that the mean HbA1c of children with DM over the last 15 years was 9.07%, and countries with higher income were significantly associated with better glycemic control than those with lower incomes. Nearly half of the diabetic children’s HbA1c were ≥9.0%, and only about a quarter of the children attained the glycemic goal of HbA1c <7.5%.

In addition to the DCCT/EDIC and UKPDS study that demonstrated long-term health benefits of strict glycemic control in adults with DM (8, 9), studies in children also showed that tighter glycemic control (represented by lower HbA1c) is associated with less premature death and increased life expectancy (7, 28). In addition, a lower HbA1c concentration is also associated with improved quality of life and less diabetic complications (29, 30). The previously less strict glycemic control recommendations in children were largely a trade-off between these long-term benefits and the children’s proneness to intensive-treatment related hypoglycemia when compared to adults (31). Owing to the increased accessibility to anti-diabetic advancements such as insulin analogs, continuous subcutaneous insulin infusion, and continuous glucose monitoring devices that improve glycemic control with minimal effect on increasing the risk of hypoglycemia in children, there is a trend for establishing stringent glycemic control goals in recent international guidelines (32, 33). Nevertheless, it remained important to individualize the designation of glycemic goals considering the patient’s hypoglycemia awareness, history of severe hypoglycemia, and compliance with therapy (16, 34).

The income level of a country closely correlates with factors directly affecting glycemic control, such as patients’ socioeconomic status, and access to healthcare resources (17, 18). However, our results showed that the effect of the country income level on HbA1c was primarily seen in T1DM, whereas HbA1c in T2DM was less influenced by country income level. In LICs, HbA1c is even higher in younger patients with short disease duration than the remaining children population. These findings support the speculation that lack of access to intensive diabetes healthcare resources could be the major obstacle to glycemic control in LICs. Recent advancements in anti-diabetic technology allows patients in HICs to reach tighter glycemic control goals, these obstacles need to be eliminated before patients in middle- and low-income countries can improve their glycemic control. In HIC, UMIC, and LMIC, diabetic children in late puberty or with longer disease duration are related to higher HbA1c, suggesting that these patients can be targeted subgroups of the pediatric population to further improve glycemic control. Similar to previous studies, our results showed that internal migration, probably reflecting less healthcare insurance and lower social economic status in the family, was associated with higher HbA1c in children (17, 18). The current study also showed that children in middle- and low income countries, which comprise 90% of the world’s children population (35), made up <10% of the population in literature on pediatric DM studies. In addition to the pursuit of social–economic development, there is a need for more DM studies on children in middle- and lower-income countries to help identify the target patient population for further healthcare improvement (36).

There are substantial limitations in our study. First, the use of historical data from hospitals and literature reflects a narrow view of the whole pediatric DM population. It can be biased when compared to population-based surveys in many domains, including selection bias from tertiary hospitals, the likely incomplete data on the HbA1c status (especially among migrant patients), and the use of household registration address can be biased from true migration status of the patients. Second, both hospital records and literature review are at risk of “survivorship bias”, which can be reflected in our literature review which shows that most studies are conducted in HICs. Patients in LICs and those with the highest HbA1c are likely to be neglected (20); hence, there is a possibility that the actual glycemic control worldwide is worse than our estimation. Third, the statistical method and assumption also created a bias from missing data imputation, distortion of originally skewed distribution (arithmetic mean is usually higher than median in the distribution of HbA1c data), and limited patient number in LICs (37). Nevertheless, our results were similar to previous large-scale studies in that the average HbA1c in children in HICs is about 8.0% (12). We believe our results can be used as a proximation to the real-world HbA1c distribution and help in the initiation of future in-depth investigations of DM in children, especially in middle- and low-income countries.

## Conclusions

In the past decade, middle- and low income countries together contributed to a quarter of the published literature (6.1% of the subjects) on HbA1c in pediatric DM. Data from these studies showed that in the past 15 years, there was an average of 76.4% children with DM worldwide who could not attain the glycemic control goal of HbA1c <7.5%. Even in HICs, there were still 67.6% children with DM who did not have optimized HbA1c. The glycemic control has improved in east China in the recent decade. Worse glycemic control was observed among migrant patients than among resident patients. There is substantial room for improvement in glycemic control in children with DM worldwide. 

## Data Availability Statement

The raw data supporting the conclusions of this article will be made available by the authors, without undue reservation.

## Ethics Statement

The studies involving human participants were reviewed and approved by the Faculty Hospital Ethics Committee in Children’s Hospital of Fudan University (2015 [49]) Ethics Committee of Children’s Hospital of Soochow University (2014KS010). Written informed consent to participate in this study was provided by the participants’ legal guardian/next of kin.

## Author Contributions

XC contributed to study design and conception, systematic review of literature, gathering patients’ data, and result interpretation. ZP contributed to systematic review of literature, gathering patients’ data, data analysis, and result interpretation. ZX, MZ, ZZ, and WL contributed to gathering patients’ data, and results interpretation. LC, FL, and TC contributed to study design and conception, and results’ interpretation. CS contributed to study design and conception, systematic review of literature, data acquisition, results’ interpretation, and manuscript drafting. All authors contributed to the article and approved the submitted version.

## Funding

The study received financial support from the National Key Research and Development Program of China (2016YFC1305302, FL), Children’s Hospital of Fudan University (EK112520180305, CS), and Suzhou Science and Technology Development Project (SS202064, TC).

## Conflict of Interest

The authors declare that the study was conducted in the absence of any commercial or financial relationships that could lead to potential conflict of interest.
