# Corrigendum: Glycated Hemoglobin (HbA1c) Concentrations Among Children and Adolescents With Diabetes in Middle- and Low-Income Countries, 2010–2019: A Retrospective Chart Review and Systematic Review of Literature

**DOI:** 10.3389/fendo.2021.714389

**Published:** 2021-09-20

**Authors:** Xiuli Chen, Zhou Pei, Miaoying Zhang, Zhenran Xu, Zhuhui Zhao, Wei Lu, Linqi Chen, Feihong Luo, Ting Chen, Chengjun Sun

**Affiliations:** ^1^Department of Endocrinology, Genetics and Metabolism, Children’s Hospital of Soochow University, Suzhou, China; ^2^Department of Pediatric Endocrinology and Inherited Metabolic Diseases, Children’s Hospital of Fudan University, Shanghai, China

**Keywords:** glycemic control, diabetes mellitus, childhood, HbA1c, middle- and low-income country

## Error in Figure/Table

In the original article, there was a mistake in **Table 1** *| HbA1c in childhood DM patients in east China* as published. There were mistakes in the calculation of percentage in lines “Proportion of HbA1c <7.5%” and “Proportion of HbA1c ≥9.0%” and in column “Resident” and “Migrant”. The corrected **Table 1** *| HbA1c in childhood DM patients in east China* appears below.

**Table 1:** HbA1c in childhood DM patients in east China.

	All	Patient type		P-value^*^
		Resident	Migrant	
Number of patients/HbA1c measures	843/2658	403/1473	440/1185	
T1DM, N (%)	791 (93.8%)	373 (92.6%)	418 (95%)	0.62
Age	11.2 ± 3.9	11.3 ± 3.8	11.1 ± 4	0.049
Male, N (%)	407 (48.3%)	191 (47.4%)	216 (43.4%)	0.81
Duration of diabetes	4.4 ± 2.8	4.5 ± 2.8	4.2 ± 2.8	<0.001
Follow-up duration	2.1 ± 2.0	2.3 ± 2.1	2.0 ± 1.9	<0.001
Number of HbA1c measures per patient	3.2 ± 3.3	3.7 ± 3.8	2.7 ± 2.6	<0.001
Number of HbA1c measures per patient-year	1.8 ± 1.3	1.8 ± 1.1	1.7 ± 1.4	0.016
HbA1c	8.1 ± 2.2	7.9 ± 2	8.4 ± 2.4	<0.001
Proportion of HbA1c <7.5%	1267 (47.7%)	759 (51.5%)	508 (42.9%)	
Proportion of HbA1c ≥9.0%	685 (25.8%)	304 (20.6%)	381 (32.2%)	
DKA events				
total events (events per 1,000 patient-years)	45 (25)	18 (20)	27 (31)	
*HbA1c in subgroups*				
by diabetes type				
T1DM	8.1 ± 2.2	7.9 ± 2.0	8.4 ± 2.4	<0.001
T2DM	7.7 ± 2.8	7.4 ± 2.6	8.1 ± 3.0	0.13
by sex				
Male	8.1 ± 2.2	7.8 ± 1.9	8.4 ± 2.4	<0.001
Female	8.2 ± 2.3	8.0 ± 2.1	8.4 ± 2.4	0.002
by age				
0-3y	7.8 ± 1.6	7.5 ± 1.1	7.9 ± 1.8	0.86
3-9y	7.6 ± 1.6	7.4 ± 1.3	7.8 ± 1.9	0.11
9-15y	8.3 ± 2.3	8.0 ± 2.1	8.6 ± 2.5	<0.001
15-18y	8.8 ± 2.8	8.6 ± 2.7	9.2 ± 2.9	0.02
by year				
2010-2014	8.5 ± 2.4	8.3 ± 2.2	8.7 ± 2.5	0.005
2015-2019	8.0 ± 2.2	7.8 ± 2.0	8.2 ± 2.4	0.004

Data are presented in mean ± standard deviation, DM, diabetes mellitus; HbA1c, glycated hemoglobin; *comparison between resident and migrant patients.

The authors apologize for this error and state that this does not change the scientific conclusions of the article in any way. The original article has been updated.

## Publisher’s Note

All claims expressed in this article are solely those of the authors and do not necessarily represent those of their affiliated organizations, or those of the publisher, the editors and the reviewers. Any product that may be evaluated in this article, or claim that may be made by its manufacturer, is not guaranteed or endorsed by the publisher.

