# Supplementary material for: Glycated Hemoglobin (HbA1c) Concentrations Among Children and Adolescents With Diabetes in Middle- and Low-Income Countries, 2010–2019: A Retrospective Chart Review and Systematic Review of Literature
Source: Front Endocrinol (Lausanne). 2021 Apr 12;12:651589. doi: 10.3389/fendo.2021.651589 (PMC8072468; doi:10.3389/fendo.2021.651589)

**Glycated hemoglobin (HbA1c) concentrations among children and adolescent with diabetes in middle- and low-income countries, 2010–2019: retrospective chart review and systematic review of literature**

**Supplementary files**

**Supplemental table 1 |** Search syntax and outcome (Ovid Medline)

| Searches | Syntax | Results |
| --- | --- | --- |
| #1 | diabetes.ti. | 233482 |
| #2 | limit 1 to yr="2010 - 2020" | 119922 |
| #3 | insipidus.ti,ab. | 8730 |
| #4 | animal/ not human/ | 4728825 |
| #5 | (child$ or adolescen$).ti,ab. | 1557558 |
| #6 | or/3-4 | 4736516 |
| #7 | 2 not 6 | 113113 |
| #8 | 7 and 5 | 8645 |
| #9 | limit 8 to ("review" or "comment") | 1199 |
| #10 | 8 not 9 | 7446 |

**Supplemental table 2 |** Number of patient simulations using published data between 2005 and 2019

|  |  |  |  | *By income* | *category* |  |
| --- | --- | --- | --- | --- | --- | --- |
|  | All |  | HIC | UMIC | LMIC | LIC |
| Total N | 487,046(484,897-489,306) |  | 457,362 (455,095-459,854) | 18,808 (18,325-19,469) | 9,376 (9,078-9,632) | 1,456 (1,354-1,605) |
| *By diabetes type* |  |  |  |  |  |  |
| T1DM | 481,802 (479,488-484,000) |  | 452,426 (450,006-454,833) | 18,617 (18,131-19,273) | 9,303 (9,008-9,564) | 1,412 (1,308-1,566) |
| T1DM | 2,378 (2,298-2,449) |  | 2259 (2,180-2,326) | 26 (23-30) | 84 (72-92) | 9 (6-13) |
| *By gender* |  |  |  |  |  |  |
| Male | 248,812 (247647-250356) |  | 234,617 (233,484-236,262) | 8,956 (8,676-9,232) | 4,520 (4,397-4,690) | 700 (638-798) |
| Female | 238,234 (236917-239321) |  | 222,744 (221,480-223,881) | 9,852 (9,569-10,294) | 4,856 (4,665-4,950) | 756 (709-808) |
| *By age* |  |  |  |  |  |  |
| 1-3y | 815 (738-865) |  | 750 (678-801) | 34 (25-41) | 30 (22-34) | NA |
| 3-9y | 59,194 (58,870-59,544) |  | 54,834 (54,575-55,149) | 2,699 (2,601-2,867) | 1,553 (1,508-1,595) | 98 (59-168) |
| 9-15y | 285,763 (284,310-287,458) |  | 267,644 (266,056-269,545) | 11,518 (11,216-11,960) | 5,678 (5,426-5,848) | 898 (795-995) |
| 15-18y | 141,274 (140,562-141,743) |  | 134,133 (133,426-134,678) | 4,557 (4,413-4,733) | 2,116 (2,016-2,207) | 460 (364-537) |
| *By year* |  |  |  |  |  |  |
| 2005-2009 | 114,703 (104,349-126,393) |  | 105,501 (95,291-117,478) | 5,897 (5,545-6,308) | 2,867 (2,657-3,129) | 394 (327-496) |
| 2010-2014 | 245,846 (236,024-255,158) |  | 233,150 (223,580-242,572) | 8,503 (8,267-8,763) | 3,518 (3,422-3,674) | 676 (627-727) |
| 2015-2019 | 126,497 (125,254-128,280) |  | 118,711 (117,517-120,384) | 4,409 (4,199-4,529) | 2,992 (2,915-3,057) | 386 (366-426) |

Data are presented in median (95% confidence interval), DM, diabetes mellitus, HIC, high-income country; UMIC, upper middle-income country; LMIC, lower middle-income country; LIC, low-income country; N, number; T1DM, type 1 diabetes mellitus; T2DM type 2 diabetes mellitus; NA, not available.

**Supplemental table 3 |** Sensitivity analysis (summary of simulations between 2005 and 2019)

|  |  |  | Study | exclusion |  |  |
| --- | --- | --- | --- | --- | --- | --- |
|  | All studies | Sample size > 500 | Unknown  data year | Unknown  age | Unknown  diabetes duration | Unknown gender |
| N of included studies | 1164 | 875 | 562 | 1011 | 875 | 1085 |
| N of patient simulations | 486,416  (481,031-489,129) | 344,943  (339,013-347,089) | 383,789  (383,254-384,473) | 441,295  (435,154-443,133) | 442,145  (435,521-444,627) | 475,814  (468,351-478,900) |
| Male (%) | 48.7% (46.4-51.4%) | 48.0% (43.2-51.5%) | 48.8% (46.4-51.5%) | 48.1% (43.4-51.4%) | 47.8% (41.2-51.4%) | 48.7% (46.1-51.4%) |
| Average HbA1c (in %) | 9.07 (8.22-10.23) | 8.99 (8.26-9.82) | 9.08 (8.18-10.24) | 9.01 (8.19-9.99) | 8.95 (8.21-9.91) | 9.06 (8.21-10.24) |
| Proportion (HbA1c < 7.5%) | 23.6% (11.5-32.5%) | 24.1% (14.8-31.8%) | 23.7% (11.4-33.3%) | 24.0% (13.1-33.2%) | 24.6% (14.0-32.8%) | 23.7% (11.6-32.6%) |
| Proportion (HbA1c ≥ 9.0%) | 46.9% (28.8-69.0%) | 45.6% (29.9-62.4%) | 47.5% (27.8-70.3%) | 46.0% (28.1-66.1%) | 44.8% (28.6-64.4%) | 46.7% (28.7-69.3%) |
| *Average HbA1c in subgroups* |  |  |  |  |  |  |
| by diabetes type |  |  |  |  |  |  |
| T1DM | 9.07 (8.22-10.25) | 8.99 (8.25-9.83) | 9.08 (8.18-10.26) | 9.01 (8.19-10.02) | 8.95 (8.20-9.96) | 9.07 (8.21-10.26) |
| T2DM | 8.85 (8.09-9.87) | 8.88 (8.09-10.05) | 8.93 (7.75-9.97) | 9.01 (8.19-10.02) | 8.95 (8.20-9.96) | 8.77 (8.04-9.59) |
| by gender |  |  |  |  |  |  |
| Male | 9.06 (8.22-10.29) | 8.97 (8.25-9.87) | 9.07 (8.18-10.32) | 9.00 (8.19-10.08) | 8.94 (8.20-9.98) | 9.05 (8.21-10.27) |
| Female | 9.07 (8.23-10.22) | 9.00 (8.26-9.92) | 9.09 (8.19-10.22) | 9.02 (8.19-10.03) | 8.96 (8.21-9.98) | 9.07 (8.22-10.26) |
| by age |  |  |  |  |  |  |
| 1 – <3y | 8.57 (7.19-10.61) | 8.47 (6.72-10.68) | 8.63 (7.41-10.62) | 8.71 (7.59-11.43) | 8.47 (7.39-10.33) | 8.62 (6.88-11.17) |
| 3 – <9y | 9.10 (8.02-11.92) | 8.83 (7.97-11.16) | 9.03 (7.99-11.75) | 8.98 (7.95-11.61) | 8.85 (7.98-11.42) | 9.16 (8.01-12.55) |
| 9 – <15y | 9.08 (8.17-10.59) | 8.98 (8.23-10.11) | 9.09 (8.14-10.55) | 9.01 (8.14-10.44) | 8.95 (8.16-10.48) | 9.08 (8.17-10.71) |
| 15 – <18y | 9.08 (8.21-10.35) | 9.06 (8.24-10.25) | 9.08 (8.17-10.32) | 9.06 (8.20-10.32) | 9.03 (8.20-10.39) | 9.09 (8.21-10.35) |
| by duration |  |  |  |  |  |  |
| 1 – <6y | 9.07 (8.17-10.48) | 8.98 (8.21-9.95) | 9.06 (8.14-10.49) | 8.99 (8.15-10.16) | 8.93 (8.16-10.16) | 9.06 (8.16-10.55) |
| 6 – <12y | 9.05 (8.23-10.31) | 8.96 (8.25-10.05) | 9.11 (8.19-10.48) | 8.98 (8.2-10.19) | 8.92 (8.22-10.27) | 9.05 (8.23-10.35) |
| 12 – <18y | 9.1 (7.86-11.24) | 8.9 (7.45-11.43) | 9.17 (7.81-11.36) | 8.97 (7.55-11.58) | 8.98 (7.66-11.46) | 9.13 (7.91-11.07) |
| by year |  |  |  |  |  |  |
| 2005–2009 | 9.00 (8.14-12.10) | 8.86 (8.15-10.99) | 8.67 (7.55-10.6) | 8.86 (8.11-11.1) | 8.87 (8.11-11.21) | 8.98 (8.14-12.16) |
| 2010–2014 | 9.15 (8.11-11.10) | 8.99 (8.13-10.99) | 9.10 (8.07-10.99) | 9.13 (8.09-11.2) | 9.02 (8.09-11.24) | 9.15 (8.11-11.08) |
| 2015–2019 | 9.06 (8.09-10.47) | 8.80 (7.99-10.47) | 9.06 (8.09-10.45) | 9.02 (8.01-10.78) | 9.01 (8.00-11.02) | 9.05 (8.09-10.49) |

Results are presented in mean and 95% confidence interval of the estimation.

**Supplemental figure 1 |** HbA1c (grey dots) in children with DM in the current study (east China, 2010 to 2020), and HbA1c trend assessed by linear regression (black solid line).


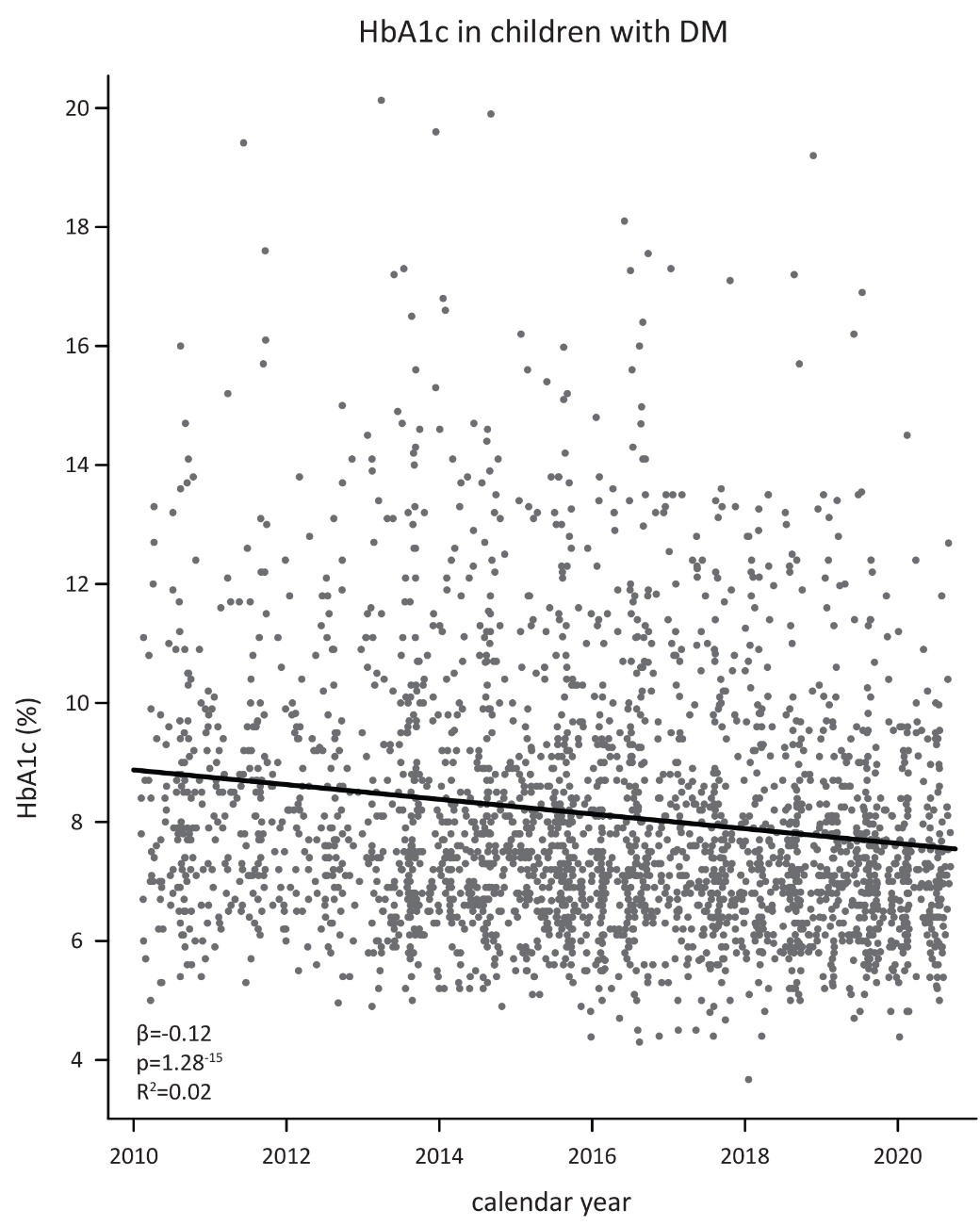

Supplement: Supplementary file 1 [file DataSheet_1.docx]
